# Supplementary material for: Seroprevalence of COVID-19 infection among vaccine naïve population after the second surge (June 2020) in a rural district of South India: A community-based cross-sectional study
Source: PLoS One. 2022 Mar 10;17(3):e0265236. doi: 10.1371/journal.pone.0265236 (PMC8912234; doi:10.1371/journal.pone.0265236)
Supplement: Attachment — Submitted filename: Response to reviewer .docx [file pone.0265236.s003.docx]

**Response to the reviewer’s comments**

1.The Methods section states that seroprevalence was quantified using an assay that measures primarily IgG and some IgA + IgM antibodies against RBD. The Abstract states that IgG and IgM antibodies against RBD were quantified. This discrepancy needs to be resolved. More importantly, can the authors provide relative abundance levels of IgG and IgM antibodies? This would for obvious reasons provide key insights into the duration of infection. These analyses should either be added, or if the data is unavailable, this should explicitly be discussed as a study limitation.

Response :

The seroprevalence was quantified using an assay that measures primarily IgG and IgM antibodies against RBD. We have made corrections in the abstract and the main article and ensured we are consistent. We don’t have data on relative abundance of IgG and IgM as the kit we have used does not give titres against each antibodies. We have discussed this as one of the limitation of the study.The kit gives a total antibody titre without relative measures of IgG, IgM antibodies. We have analysed total antibody titre for males and females and stratified by age. This finding has been presented as a graph. [line no. 125-126, 200-201, 330-334 & Fig.1)

2. The study was carried out in a population that hadn't received a COVID-19 vaccine. Given the timeline of the study, this could be either due to lack of access to a COVID-19 vaccine or general vaccine hesitancy. However, are other vaccination records available for these individuals? This information may help identify 2 sub-groups in the population - a) those that have completed recommended vaccination regimens for other vaccines i.e., they had likely not received a COVID-19 vaccine at the time of the study due to lack of access (potentially lower risk group), and b) those that had not completed recommended vaccination regimens even for other vaccines i.e., they likely have general vaccine hesitancy (potentially higher risk group). Are there indeed differences in seropositivity between these 2 groups?

Response :

The fierce delta pandemic wave in India disrupted almost everything including vaccine distribution. The study was conducted soon after this wave and people in the study did not take the vaccine due to vaccine shortage and not due to vaccine hesitancy. As a hospital, we have provided access to vaccination in these villages and all of them took vaccine in a matter of one month.

3. Table 3 provides insights into how univariate factors are associated with seroprevalence. The insights from this table are limited as the significant univariate factors are the "obvious" ones (symptoms, positive test for individual or family members). It would be much more interesting to examine the contributions of the "non-obvious" features to seroprevalence risk in a multivariate setting (potentially also accounting for interaction terms).

Response:

The study was conducted in most challenging circumstances, where the health system failed to cater to the needs of the population leading to many preventable deaths. In that situation, we limited our questions to the bare minimum. Hence we do not have any other data to examine the contributions of the "non-obvious" features to seroprevalence risk in a multivariate setting.

4. From a presentation standpoint, the manuscript would benefit significantly from some figures/visual representations that summarize the data currently presented as tables.

Response:

We have analysed total antibody titre for males and females and stratified by age. This finding has been presented as a graph
